# Identification of foot and mouth disease risk areas using a multi-criteria analysis approach

**DOI:** 10.1371/journal.pone.0178464

**Published:** 2017-05-26

**Authors:** Diego Viali dos Santos, Gustavo Sousa e Silva, Eliseu José Weber, Heinrich Hasenack, Fernando Henrique Sautter Groff, Bernardo Todeschini, Mauro Riegert Borba, Antonio Augusto Rosa Medeiros, Vanessa Bielefeldt Leotti, Cláudio Wageck Canal, Luis Gustavo Corbellini

**Affiliations:** 1 Departamento de Saúde Animal, Secretaria de Defesa Agropecuária, Ministério da Agricultura Pecuária e Abastecimento, Brasília, Brazil; 2 Laboratório de Epidemiologia Veterinária, Faculdade de Veterinária, Universidade Federal do Rio Grande do Sul, Porto Alegre, Rio Grande do Sul, Brazil; 3 Laboratório de Geoprocessamento, Centro de Ecologia, Universidade Federal do Rio Grande do Sul, Porto Alegre, Rio Grande do Sul, Brazil; 4 Departamento de Defesa Agropecuária, Secretaria da Agricultura, Pecuária e Irrigação do Estado do Rio Grande do Sul, Porto Alegre, Rio Grande do Sul, Brazil; 5 Departamento de Estatística, Instituto de Matemática e Estatística, Universidade Federal do Rio Grande do Sul, Porto Alegre, Rio Grande do Sul, Brazil; 6 Laboratório de Virologia, Faculdade de Veterinária, Universidade Federal do Rio Grande do Sul, Porto Alegre, Rio Grande do Sul, Brazil; Shanxi University, CHINA

## Abstract

Foot and mouth disease (FMD) is a highly infectious disease that affects cloven-hoofed livestock and wildlife. FMD has been a problem for decades, which has led to various measures to control, eradicate and prevent FMD by National Veterinary Services worldwide. Currently, the identification of areas that are at risk of FMD virus incursion and spread is a priority for FMD target surveillance after FMD is eradicated from a given country or region. In our study, a knowledge-driven spatial model was built to identify risk areas for FMD occurrence and to evaluate FMD surveillance performance in Rio Grande do Sul state, Brazil. For this purpose, multi-criteria decision analysis was used as a tool to seek multiple and conflicting criteria to determine a preferred course of action. Thirteen South American experts analyzed 18 variables associated with FMD introduction and dissemination pathways in Rio Grande do Sul. As a result, FMD higher risk areas were identified at international borders and in the central region of the state. The final model was expressed as a raster surface. The predictive ability of the model assessed by comparing, for each cell of the raster surface, the computed model risk scores with a binary variable representing the presence or absence of an FMD outbreak in that cell during the period 1985 to 2015. Current FMD surveillance performance was assessed, and recommendations were made to improve surveillance activities in critical areas.

## Introduction

Foot and mouth disease (FMD) is a highly contagious viral disease which affects all cloven-hoofed animals. The presence of the disease has been studied since the sixteenth century in many countries [1] and it has a negative impact on livestock productivity in countries where the disease is endemic. Moreover, in countries that have been previously free of FMD, new outbreaks result in large financial losses due to the decrease of export markets [2].

**Fig 1:**
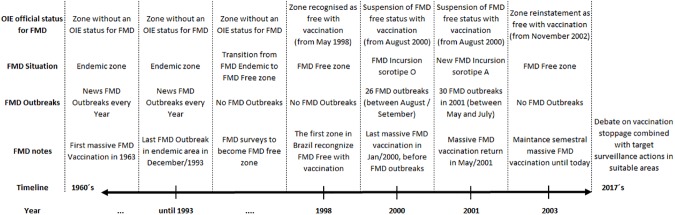
Overview of the FMD history in the state of Rio Grande do Sul / Brazil.

Up until the 1990s outbreaks of FMD were common in many countries of South America [3]. Fortunately, this scenario has changed, and there has been an absence of FMD outbreaks since 2012 on this continent [4]. An annual meeting on this subject has occurred for 44 years where specialists from these countries gather to assess and discuss the progress made by national governments. The progresses and challenges in South America’s FMD program have been discussed at great length in these seminars [5]. For instance, the cessation of FMD vaccination combined with target surveillance actions in suitable areas are one of the most important questions on this matter. Specifically, the economic consequences involved in stopping cattle vaccination and how animal products from disease-free zones without FMD vaccination may access new markets should be analyzed owing to its significant impact on the livestock industry for South American countries and other nations [6]. In the state of Rio Grande do Sul (RS), which is the region encompassed in this study, there is an overview of the FMD history displayed in Fig 1 [7, 8].

In this sense, risk identification methods can aid the veterinary services in developing surveillance strategies, particularly for highly transmissible diseases [9]. Spatial knowledge-driven methods, e.g., multi-criteria decision analysis (MCDA), have been used on targeted surveillance programs [10] and it could help Official Veterinary Service (OVS) to determine FMD risk areas in addition to enhancing animal health surveillance. The main advantage in using MCDA is to facilitate decision-makers learning about this theme and to have a better understanding of problem solving [11].

The aim of this study was to create a spatial knowledge-driven model based on MCDA, which may identify risk areas for FMD occurrence in RS. In addition, the current FMD surveillance strategies were evaluated and compared across identified FMD high-risk areas, and recommendations were made to improve surveillance activities in critical zones.

## Material and methods

Description of our analytical approach is divided into three main sections. In the first section we introduce the studied area. In the second one, we describe the model structure that is composed by: pathway and risk factor values and the weight of experts’ opinions; data layers; combination of pathways; validation of FMD risk maps against historical FMD outbreaks and sensitivity analysis and uncertainty. Finally, in the third section, the way in which we model the FMD surveillance performance is described.

### Study area

**Fig 2:**
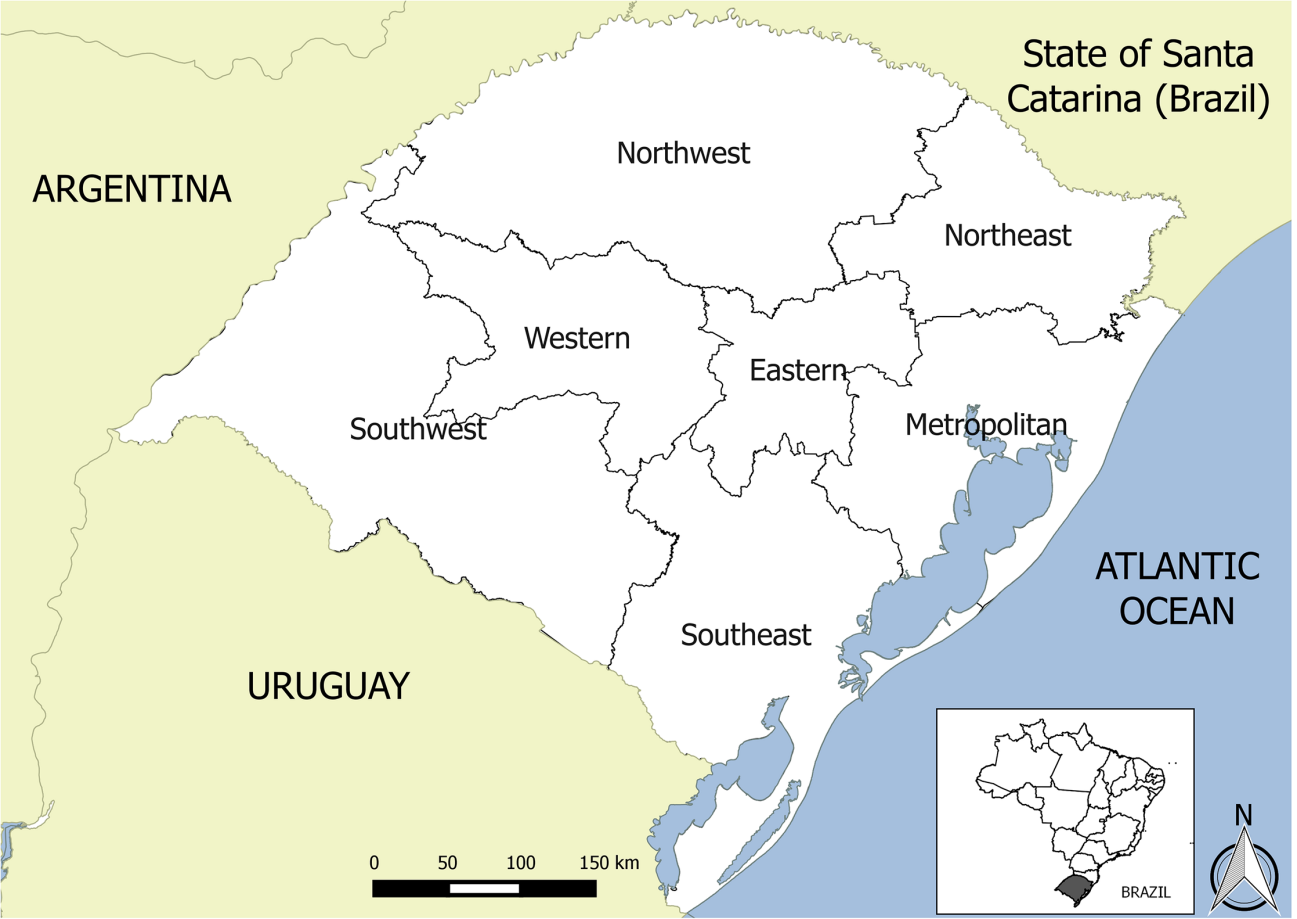
Map of Rio Grande do Sul showing the seven mesoregions and the national and international borders.

This study was conducted in the RS, Brazil, due to its importance for local agribusiness. RS has a relatively large domestic animal population comprised of approximately 14 million cattle, 6 million swine and 4 million sheep [12]. The state is comprised of a total land area of 269,000 km^2^. It is situated between the latitudes of 27°04’S to 33°45’S and the longitudes of 49°41’W to 57°38’W. The climate is humid subtropical with a relative humidity of up to 60% for most of the year. The state is divided into 7 mesoregions (Fig 2) [13]. It has an extensive and peaceful international border neighbouring Uruguay in the South (1,003 km) and Argentina in the West (724 km). Moreover, RS is bordered to the North by the Brazilian state of Santa Catarina and to the East by the Atlantic Ocean (Fig 2).

### Model structure

A knowledge-driven spatial model, based on MCDA, was built using Idrisi 17.0 Selva GIS and Image Processing Software (Clark Labs, Worcester, USA). In our study, data sources were used to generate raster layers with a resolution of 1 km x 1 km or polygon layers. The list, sources, type of data and variables used in this study are presented in the Table A in S1 File.

The process of FMD occurrence in RS was divided into four steps [10], as follows:

- Introduction–the entry of the FMD virus into RS;
- Exposure–the infection of the first FMD-susceptible animal (the amount of virus that can cause the first animal infection);
- Establishment–infection of the first group (herd) of FMD-susceptible animals; and
- Spread–infection of the second and subsequent groups of FMD-susceptible animals.

In this study, introduction and exposure were combined for analysis purposes and were named the Introduction Module, which is composed of four pathways (introduction pathways, IP; Table 1). Similarly, establishment and spread were also combined and were called the Dissemination Module, which is composed of three pathways (dissemination pathways, DP; Table 2). The Tables 1 and 2 contain details of the risk factor weightings, which will be explained later in the “Pathway and risk factor values and the weight of expert opinions” section.

**Table 1:** Introduction module: Non-negligible FMD introduction pathways in Rio Grande do Sul, Brazil and their weighting values as given by expert opinions.

Pathway code	Pathway name	Description pathway	Weighting average (min; max)
IP1	Illegal live animals	Importation of live animals through unofficial channels, avoiding previous tests and quarantine measures	0.503 (0.390; 0.604)
IP2	Illegal animal products	Importation of products derived from animals, via unofficial channels, avoiding previous tests and quarantine measures	0.275 (0.200; 0.390)
IP3	Fomites	Entry of potentially FMD virus contaminated clothing, footwear, vet and others equipment, or other non-animal commodities.	0.151 (0.063; 0.210)
IP4	Bioterrorism	Intentional importation of infectious material and dispersal into FMD susceptible animals.	0.071 (0.039; 0.150)
Total weighting of the introduction pathways (sum of the average)			1

**Table 2:** Dissemination module: Non-negligible FMD dissemination pathways in Rio Grande do Sul, Brazil and their weighting values as given by expert opinions.

Pathway code	Pathway name	Description pathway	Weighting average (min; max)
DP1	Direct contact	FMD virus transmitted from animal to animal via close contact, either within a herd, between herds on neighboring properties, or when infected animals are moved from one location to another	0.620 (0.405; 0.731)
DP2	Fomites	FMD virus able to survive in the environment and be moved from farm to farm on contaminated equipment, vehicles or animal products	0.274 (0.143; 0.481)
DP3	Windborne	FMD virus able to survive in the environment and be spread from farm to farm by wind in the absence of close contact between animals	0.106 (0.072; 0.143)
Total weighting of the dissemination pathways (sum of the average)			1

The possible FMD introduction and dissemination pathways were established after analyzing all the FMD outbreak investigations reported worldwide between 1996 and 2012 and published on OIE’s website [8, 14]. Moreover, other FMD outbreak publications [15–17] and unpublished data from the Brazilian OVS were evaluated. In order to facilitate this analysis, a scenario tree showing the FMD introduction pathways was built and it is presented in the Scenario tree in S1 File

FMD introduction through legal products and animal movement via legal importation of live animals, animal products, genetic materials, vaccines or biological agents were considered pathways with a negligible likelihood and were not considered further in the analysis due to laboratory tests and quarantine routinely executed according to the Brazilian Veterinary Surveillance Program for Vesicular Diseases [18]. FMD introduction through wildlife was considered a pathway with negligible likelihood because it has never been reported in South America, beyond the fact that the main wildlife species involved with FMD outbreaks are exotic and only exist on the continent in captivity [19]. Despite several FMD outbreaks as a result of laboratory escapes (15, 17), this pathway was considered to have negligible likelihood because the manipulation of the FMD virus in RS laboratories has been prohibited since 1994 [20].

For each pathway in each module, several risk factors (RF) were described (Tables 3 and 4). Risk factors are the variables which might be associated with FMD introduction and dissemination. The variables were identified through a literature review (unpublished data from the Brazilian OVS; [2, 10, 21] and were considered for building the spatial data layers.

**Table 3:** Risk factors of the FMD introduction pathways in Rio Grande do Sul, Brazil and their weighting values as given by expert opinions.

Pathway associated	Risk factor name	Reason for using	Weighting average (min; max)
IP1	International border proximity	The difference in price (exchange) between the countries can improve an illegal flow of animals, especially cattle, between RS, Brazil, Argentina and Uruguay	0.484 (0.153; 0.639)
IP1	Bovine and buffalo count	The FMD virus exposure into RS should occur most likely between bovine and buffalo because these were the species more illegal movement animal associated	0.227 (0.096; 0.657)
IP1	Ruminant density	Ruminant species live together at pasture and may be available to FMD virus	0.165 (0.053; 0.250)
IP1	FMD susceptible animals farm density	The concentration of FMD susceptible animal farms in RS areas could help in exposure of FMD virus to FMD susceptible animal	0.124 (0.075; 0.209)
IP2	Non-commercial pig farms density	These farms have not biosecurity measures, and the pigs could be available to exposure FMD virus by equipment, vehicles or animal products contaminated	0.345 (0.100; 0.750)
IP2	Most intensive surveillance areas count	These areas have not biosecurity measures, and animals could access residue of illegal animal products to feed animals (swine)	0.655 (0.250; 0.900)
IP3	International border proximity	The people movement in international border is constant, with farmers who have farms in two countries (Brazil and Argentina or Uruguay)	0.507 (0.405; 0.637)
IP3	International ports and airports presence	The movement in areas with ports and airports can improve the likelihood of FMD introduction/exposure	0.376 (0,250; 0.481)
IP3	FMD susceptible animals farm density	Improve the likelihood of exposure FMD contaminated clothing, footwear, vet and other equipment, or other non-animal commodities with FMD susceptible animal	0.117 (0.078; 0.200)
IP4	Presence of international farms fair and waiting place for cattle export	The intentional introduction of FMD can have a severe impact if incursion occurs at sites with high concentrations of susceptible animals	0.568 (0.125; 0.875)
IP4	Ruminant farms density	The intentional introduction of the FMD virus may be areas with high concentration ruminant farms.	0.432 (0.125; 0.875)

The sum of each weighting individual introduction pathway (IP1, IP2, IP3 and IP4) should be 1 [22].

**Table 4:** Risk factors of the FMD dissemination pathways in Rio Grande do Sul, Brazil and their weighting values as given by expert opinions.

Pathway associated	Risk factor name	Reason for using	Weighting average (min; max)
DP1	Pigs movements for all purposes, except slaughter	The pigs’ movements to others farms can facilitate the dissemination by direct contact	0.221 (0.089; 0.313)
DP1	Ruminant movements for all purposes, except animal fair and slaughter	The bovine, ovine, caprine and buffalo movement between different farms can improve the likelihood of dissemination.	0.211 (0.146; 0.260)
DP1	Ruminant movements to animal fairs	Animal fairs are a good place to direct contact between ruminant animals from different farms	0.183 (0.128; 0.249)
DP1	Ruminant density	Ruminant species live together at pasture and can be available to FMD virus dissemination.	0.133 (0.103; 0.171)
DP1	Pig farms density	High concentration pig farms areas (commercial and non-commercial) may facilitate the dissemination by direct contact	0.117 (0.032; 0.229)
DP1	FMD susceptible animal movement to slaughterhouse	FMD infected animals sent for slaughter could infect other FMD susceptible animals in the slaughterhouse neighborhood by direct contact	0.111 (0.065; 0.202)
DP1	Wild boar area proximity	FMD virus could be disseminated by live wild boar	0.024 (0.012; 0.031)
DP2	Environmental suitability for FMD virus	The water droplets presence in the air can help the resistance FMD virus in fomites and in the air.	0.318 (0.074; 0.556)
DP2	Non-commercial pig farms density	These farms have not biosecurity measures and FMD virus could disseminate by equipment, vehicles or animal products contaminated	0.294 (0.058; 0.550)
DP2	Milk cattle farms, reproduction cattle farms and commercial pigs farm density	These properties using various reproductive and production techniques that increase the chance of contact FMD virus by equipment, vehicles or animal products contaminated	0.200 (0.064; 0.300)
DP2	Ruminant density	High ruminant concentration can improve likelihood to contact FMD virus by equipment, vehicles or animal products contaminated	0.188 (0.122; 0.300)
DP3	Non-commercial pig farms density	These farms have not biosecurity measures and the pigs could be an amplifying effect with a high rate of FMD virus excretion and can facilitate the dissemination by Windborne	0.338 (0.088; 0.577)
DP3	Environmental suitability for FMD virus	The water droplets presence in the air can help the resistance FMD virus in fomites and in the air.	0.270 (0.066; 0.546)
DP3	Ruminant density	High ruminant concentration can improve likelihood to dissemination FMD virus by windborne	0.252 (0.070; 0.530)
DP3	Commercial pig farms density	These properties have biosecurity measures, though not totally prevent a possible dissemination by Windborne	0.140 (0.073; 0.250)

The sum of each weighting individual dissemination pathway (DP1, DP2 and DP3) should be 1 [22].

#### Pathway and risk factor values and the weight of expert opinions

Eighteen different RF associated with the introduction and dissemination pathways and the four other RF were used to design the FMD Surveillance Performance (SP). In the Table A in S1 File, a list of RF and their relative importance in the model are shown.

For the expert eliciting process, we used a snowball sampling process [23] to select the FMD experts. This study considered an FMD expert as a professional who had at least one of the following criteria:

1. At least one publication on FMD in the past 10 years in an indexed journal;
2. Worked in the management of at least one FMD outbreak in South America in the past 30 years;
3. Experience in international organizations related to FMD control, eradication and prevention; and
4. Experience in the control, eradication and prevention of FMD in the OVS.

The initial group of FMD experts who participated in the snowball sampling was elected in a meeting with the Chief Veterinary Officer of the Ministry of Agriculture, Livestock and Food Supply for RS and the Chief Veterinary Officer of the Secretariat of Agriculture, Livestock and Irrigation of RS. FMD experts meeting the criteria were contacted by email and invited to indicate up to three FMD experts to take part in the study, who should also be selected according to the previous mentioned criteria. The experts indicated by the first group of experts received 10 matrix files (Microsoft Excel 2010^®^), which were grouped into three main categories: 1) introduction pathways and risk factors; 2) dissemination pathways and risk factors, and 3) FMD Surveillance Performance. These experts were requested to provide a weight for each risk factor (using the methodology described below) and pathways using matrices that were previously tested by two non-participating FMD experts.

The weighting procedure follows the algorithm developed by Saaty [22] under the analytical hierarchy process (AHP), which is performed through a series of pairwise comparisons of the relative importance of the factors and pathways. These pairwise comparisons were analyzed to produce a set of weights that sum to 1. Since the complete pairwise comparison matrix contains multiple paths by which the relative importance of the criteria can be assessed, it is also possible to determine the degree of consistency that has been used in developing the ratings. Saaty [22] indicates the procedure by which an index of consistency, known as a consistency ratio (CR), can be produced. The CR indicates the probability that the matrix ratings were randomly generated. Saaty indicates that matrices with CR ratings greater than 0.10 should be re-evaluated. In our study, only matrices with CRs equal to or lower than 0.10 were considered [22]. The average weight value of all the risk factors and pathways for all the experts were used as an input for the model. By the end of this stage, a Delphi-like approach [24] was applied by e-mail to obtain feedback from the experts by providing an anonymous summary of the average results of the group and allowing them to revise their individual evaluation [25]. Copies of the invitation letters to the experts and the matrix files are available from the author by request.

The results of the weighting procedure can be found in Tables 1, 2, 3 and 4.

The model was composed of layers in which the values can be spatially evaluated and compared. These layers represent the combination of the risk factors values and weights with the associated IP and DP, which were weighted by the expert opinion approach.

#### Data

The initial datasets were plotted as points or polygons in Idrisi 17.0 Selva depending on the particular dataset available. The majority of the animal data were obtained from the regional OVS and the literature [26–28]. The whole list of data layers used and their sources are included in the Table A in S1 File. Each vector map was converted to the raster format using a 1 km × 1 km spatial resolution. The proximity of the international border map was created with Idrisi 17.0 Selva by first calculating the distance between each grid and nearest international boundary, then this distance was used to create a variable (ranging from 0–1) where the shortest distance is more susceptible to FMD introduction by the border.

#### Combination of pathways

The methodology used in this study to combine the RF and pathways and obtain the likelihood of FMD occurrence was described by East et al. [10]. Afterward, the likelihood of introduction was calculated separately from the likelihood of dissemination. The two measures were subsequently combined multiplicatively, resulting in the likelihood of FMD occurrence.

The contributions of the data layers to the likelihood scores for the introduction and dissemination pathways were considered to be additive (Eq 1 and Eq 2). In each grid cell of the likelihood maps, the likelihood score for a given pathway was compiled by combining the data values for each RF, which were weighted by its assigned importance weighting (Eqs 1 and 2). Within each data layer, the highest score was classified as 1 (e.g., number of cattle movement, proximity to an international border, etc.) and the others were adjusted on a linear scale from 0 to 1 in relation to the highest value (adapted from East et al. [10]).

$${IP}_{j}=\sum_{i=1}^{I_{j}}\left({RFI}_{ij}\times {W\_RFI}_{ij}\right)$$ (1)

where *IP* represents the likelihood score for the *j*-th IP; *j* = 1,…,4; *RFI*_*ij*_ is the applicable value for the *i-th* of *I*_*j*_ RF variables applying to IP *j*, and *W_RFI*_*ij*_ is the *i-th* RF relative importance weighting within pathway *j*.

$${DP}_{l}=\sum_{k=1}^{K_{l}}\left({RFD}_{kl}\times {W\_RFD}_{kl}\right)$$ (2)

where *DP*_*l*_ represents the likelihood score for the *l*-th DP, *l* = 1,…,3; *RFD*_*kl*_ is the applicable value for the *k-th* of *K*_*l*_ RF variables applying to DP *l*, and *W_RFD*_*kl*_ is the *k-th* RF relative importance weighting within pathway *l*.

Once the likelihood score for the individual pathways was calculated, a likelihood score for the introduction module (IM) and dissemination module (DM) was calculated as the sum of the likelihood scores for each of the four introduction (*j* pathways) and of the three dissemination (*l* pathways) pathway multiplied by their weights (Tables 1 and 2) to reflect the relative importance of the pathway (Eqs 3 and 4).

$$IM=\sum_{j=1}^{4}\left({IP}_{j}\times {W\_IP}_{j}\right)$$ (3)

where *IM* represents the likelihood score for the introduction module; *IP*_*j*_ is the result of Eq 1 and *W_IP*_*j*_ is its relative importance weighting within pathway *j*.

$$DM=\sum_{l=1}^{3}\left({DP}_{l}\times {W\_DP}_{l}\right)$$ (4)

where *DM* represents the likelihood score for the dissemination module; *DP*_*l*_ is the result of Eq 2 and *W_DP*_*l*_ is its relative importance weighting within pathway *l*.

The FMD occurrence likelihood score (Eq 5) was calculated as the product of the likelihood score for the FMD introduction module and dissemination module because any given grid cell will require both a means of introduction and dissemination to propagate an FMD outbreak.

LO=IM×DM (5)

where *LO* represents the likelihood score for FMD occurrence; *IM* is the result of Eq 3 and *DM* is the result of Eq 4.

### Validation of FMD risk maps against historical FMD outbreaks

Data on RS FMD outbreaks from 1986 to 2015 were provided by Panaftosa (unpublished data). We employed the relative operating characteristic (ROC) curve to assess the model validity using data matching of identified FMD high-risk areas in comparison with the observed outbreaks. ROC curves were made in Idrisi 17.0 Selva. For this, two vectors are created, in which the first vector we calculated risk scores for each cell of the raster surface and the second vector comprised of a series of 1 and 0, denoting the presence or absence of FMD outbreak, respectively, in each grid. In this model validation, the areas considered FMD high-risk areas were the areas with a likelihood of FMD occurrence within the top 20% among the 1 km x 1 km grids, which produced the highest area under the curve (AUC) values when compared with last FMD outbreaks in RS. Two ROC curves were produced: one including the data on all outbreaks and the other with only the last outbreak (2001) using five equal-interval thresholds.

### Sensitivity analysis and uncertainty

The sensitivity analysis was conducted according to methods published by East et al. [10]. The sensitivity analysis output was the comparison between the FMD risk rankings of the seven mesoregions in RS [13] as measured by the average FMD occurrence likelihood score of the grid squares in each mesoregion.

Regarding the uncertainty analysis, the origin, inner features and type of data for all the variables were initially analyzed, and those that could have a high level of epistemic uncertainty were chosen, i.e. the ones not measured with a quantity sufficiently accurately. Subsequently, the weightings of the individual data variables were increased and decreased individually utilizing the maximum and minimum value given by the FMD experts and the effects on the final outcome were determined. Finally, individual variables were removed from the model, and the mesoregion of FMD risk rankings were compared.

### Modeling of the FMD surveillance performance

FMD surveillance performance was modeled by the usage of four variables and their weights, as evaluated by the FMD experts. These variables were “private veterinarian density”, “number of FMD-susceptible animal disease investigation reports”, “number of non-FMD-susceptible animal disease investigations reports” and “OVS presence”. An index was created (Eq 6) to evaluate FMD surveillance performance (*SP*_*index*_) [10].

$$SP_{index}=(PVD\times W_{1})+(IFMD\times W_{2})+(INFMD\times W_{3})+(OVS\times W_{4})$$ (6)

where PVD = private veterinarian density; IFMD = relative number of FMD-susceptible animal disease investigation reports; INFMD = relative number of non-FMD-susceptible animal disease investigations reports and OVS = presence of Official Veterinary Service. *W*_*n*_ represents the weightings of the relative importance of the *n-th* surveillance element as evaluated by the FMD experts, *n* = 1,…,4.

We assumed that the likelihood of FMD occurrence could be minimized by the *SP*_*index*_, which would remain a residual FMD likelihood, called residual risk [10]. We calculated this residual risk as follows (Eq 7):

$$ReR=LO\times (1-SP_{index})$$ (7)

where *ReR* is the residual risk; *LO* is the result of Eq 5; and *SP*_*index*_ is the result of Eq 6.

Finally, we compared the FMD occurrence likelihood map and residual risk map.

## Results

### Previous FMD outbreaks analysis and variables selection

The OIE database reports 57,602 FMD outbreaks in 99 countries from 1996 until 2012. Bovine is the main species associated with FMD outbreaks (68%), followed by swine (22%), ovine (20%), and wild species (1%). Only 9% of all outbreaks describe the likely origin of the FMD infection. The illegal movement of animals was associated with 58% of the reported FMD outbreaks. An analysis of the OVS records (unpublished data) identified that bovine, ovine and swine were the species involved in FMD outbreaks in RS. Moreover, these data showed that FMD virus introduction and dissemination in RS was associated with the illegal and legal movement of animals. Thus, the model variables selection was based on these data and the FMD characteristics.

### Elicitation process

The initial group of 28 FMD experts was requested to participate using snowball sampling. Among these selected experts, 14 (50%) answered by e-mail and indicated 25 FMD experts to participate in an elicitation process. The 25 indicated FMD experts were contacted, and 13 (52%) of them completed the matrices and returned them by email; four (31%) experts who responded were working in Brazil, three (23%) were employed in Argentina, two (15%) work for animal health international organizations, two (15%) were in Uruguay, one (8%) was employed in Peru, and one (8%) was employed in the USA. Moreover, when analyzing the employment area, seven (55%) FMD experts have worked for OVS, two (15%) have worked in animal laboratories, two (15%) have worked in universities and two (15%) in international organizations.

Only one (8%) FMD expert revised his previous opinion after receiving the anonymous summary of the group results and consistency index. This FMD expert altered his responses in 6 of 10 matrices (60%). The FMD experts’ responses were analyzed, and only consistent answers [22] were included in the average weighting (Tables 1–4). In all 10 matrices, the average consistency rate was seven responses (out of 13), varying between three and thirteen.

### FMD risk maps

**Fig 3:**
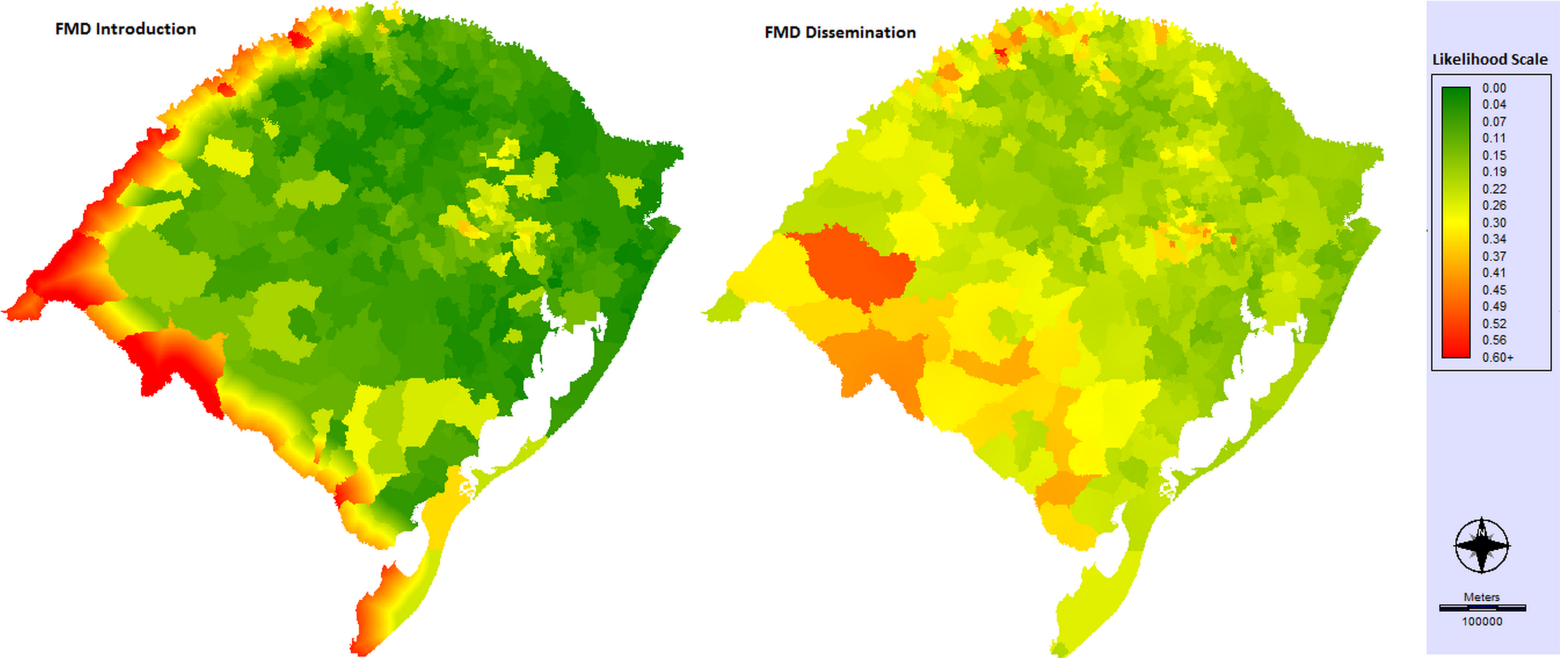
Likelihood of FMD introduction and FMD dissemination maps in Rio Grande do Sul.

The outputs (likelihood of FMD occurrence) are regional maps showing the relative likelihood of disease introduction and dissemination (Fig 3). The maps show the relative likelihood scores for each 1 km × 1 km grid cell based on the highest risk grid square on each map that was attributed the score 1. Relative likelihood is presented on a scale between 0 and 1. The maps analyses show important differences that reflect the diverse pathways of FMD entry and spread in Rio Grande de Sul.

**Fig 4:**
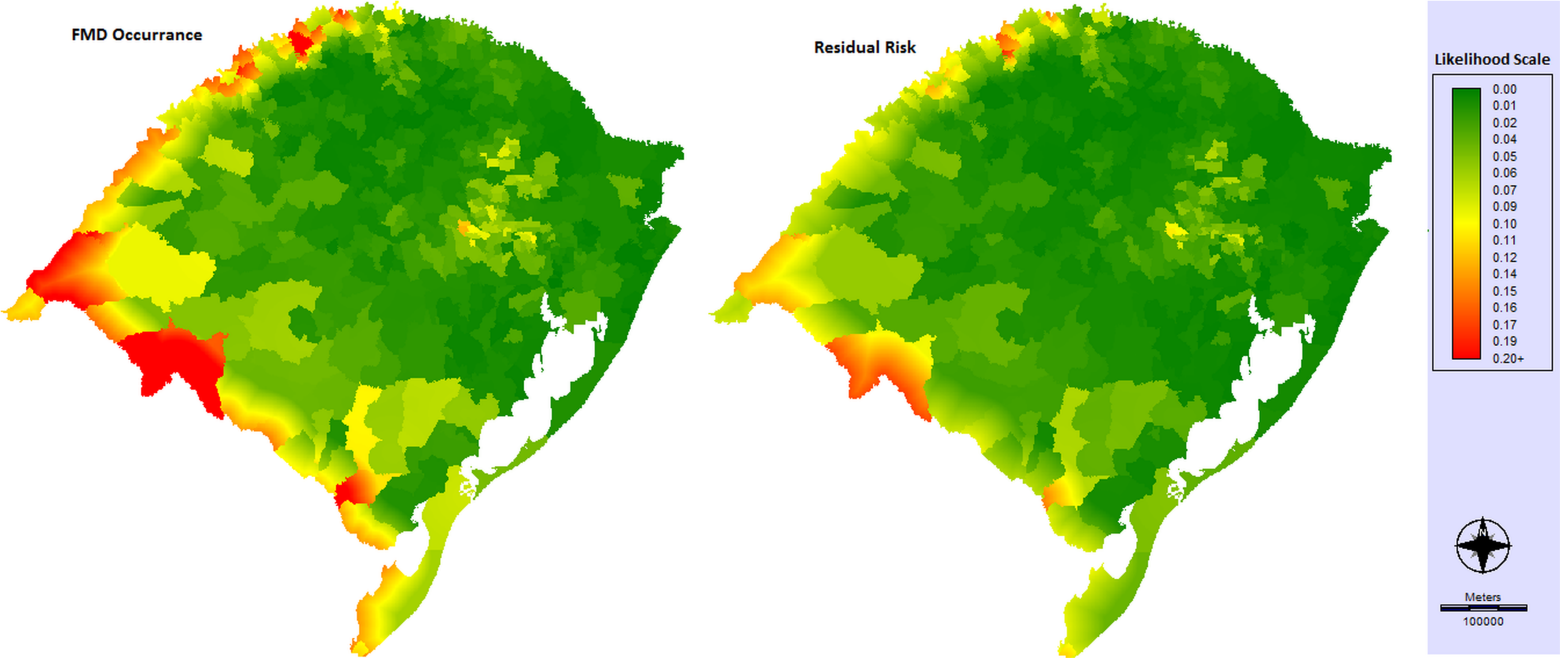
Likelihood of FMD occurrence and risk residual maps in Rio Grande do Sul.

The likelihood of FMD occurrence map is shown in Fig 4. The Southwest, Northwest and Southeast mesoregions, particularly the international border areas, and the Eastern mesoregion were identified with the presence of areas of high likelihood of FMD occurrence.

### FMD surveillance performance

The importance of each variable according to FMD was as follows: “OVS presence” 34.3%; “FMD-susceptible animal disease investigation reports” 33.7%; “density of private veterinarians” 23.7%; and “non-FMD-susceptible animal disease investigations reports” 8.3%. The *SP*_*index*_ was included into the model to verify its impact on the likelihood of FMD occurrence (Fig 4, right side and Table 5). The FMD surveillance performance had better results in the Southwest and Southeast mesoregions, which have the highest likelihood of FMD occurrence (Table 5). The final map showed the residual risk (Fig 4) with all 22 variables.

**Table 5:** FMD risk rankings for the state of Rio Grande do Sul mesoregions.

Mesoregion	Likelihood of FMD Occurrence (LO)	FMD Surveillance Performance (SP_index_)	Residual Risk (ReR)
Southwest	0.080	0.339	0.053
Southeast	0.049	0.324	0.033
Eastern	0.041	0.231	0.032
Northwest	0.037	0.232	0.028
Northeast	0.029	0.244	0.022
Western	0.020	0.307	0.014
Metropolitan	0.017	0.211	0.013

### Sensitivity analysis and uncertainty

The “proximity to international border” variable was the only risk factor that changed the mesoregion FMD risk ranking. This is the most influential variable in the model, contributing with 16% of the likelihood of FMD occurrence (Table A in S1 File), and when its weighting value increased twice, the Northwest region likelihood exceeds the Eastern. The original likelihood of FMD occurrence ranking can be visualized in Table 5. Other ranking changes did not occur, and the Southwest and Southeast have continued as the major risk areas.

The risk factors “count of most intensive surveillance areas” and “proximity to areas with presence of wild boar” were considered with a high level of uncertainty due to their data sources. OVS surveys from each county were used to gather these data; however, important information on the most intensive surveillance areas, for instance, its size (km^2^), was not collected. Moreover, considering all the variables analyzed, the “count of most intensive surveillance areas” variable was the only one with missing data (13.7%), and in these cases, the value 1 was used (the worst scenario), which caused an overestimation. This variable contributes 9% of the likelihood of FMD occurrence (Table A in S1 File). If this variable is removed from the model, a single change in the mesoregion FMD risk rankings occurs, with the Northwest mesoregion jumping from the fourth place to the second. When the maximum (0.9) and minimum (0.25) FMD expert weightings were used, no ranking changes occur. The other variable analyzed, “proximity to areas with presence of wild boar”, had a minor importance on the likelihood of FMD occurrence (less than 1%, see Table A in S1 File). The uncertainty analysis revealed that removing or varying the maximum (0.031) and minimum (0.012) FMD expert weightings for this variable did not change the mesoregion FMD risk rankings.

### Validation of the likelihood maps against historical FMD outbreaks

**Fig 5:**
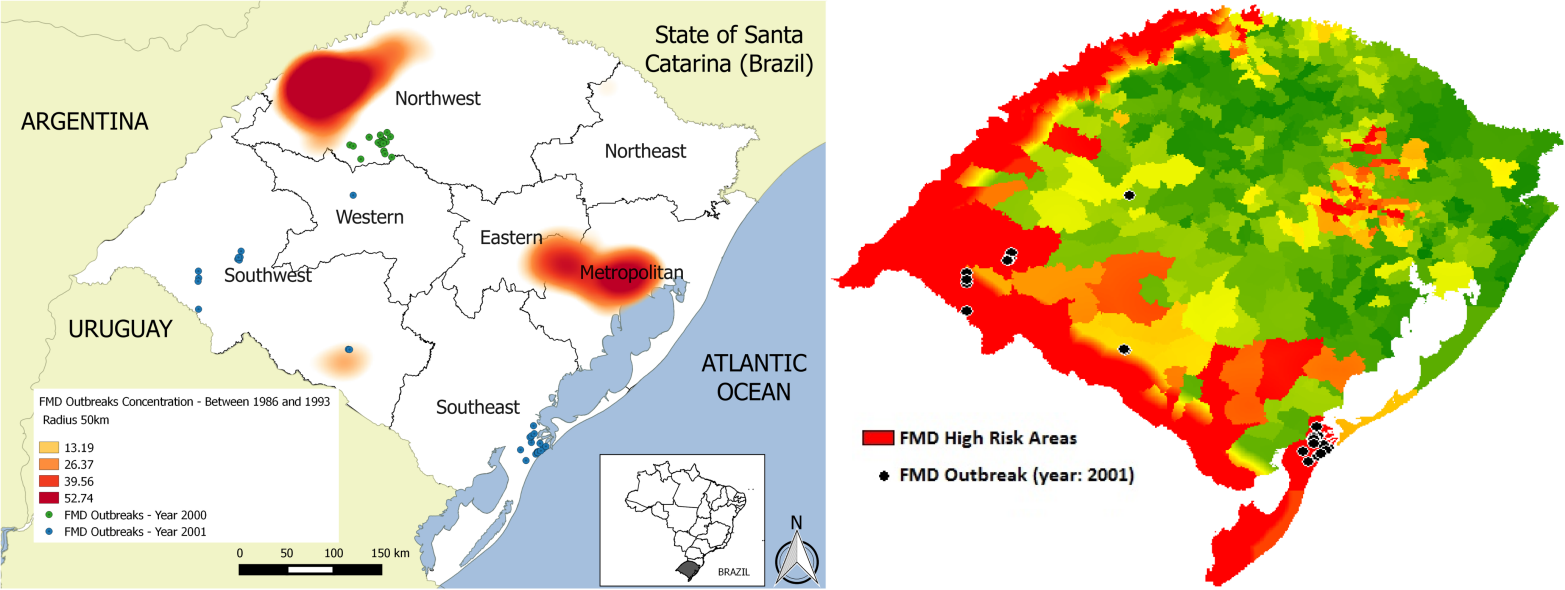
Historical FMD outbreaks in Rio Grande do Sul and FMD high-risk areas against the last FMD outbreaks in Rio Grande do Sul. *FMD outbreaks concentration: FMD outbreaks within a radius of 50 km.

**Fig 6:**
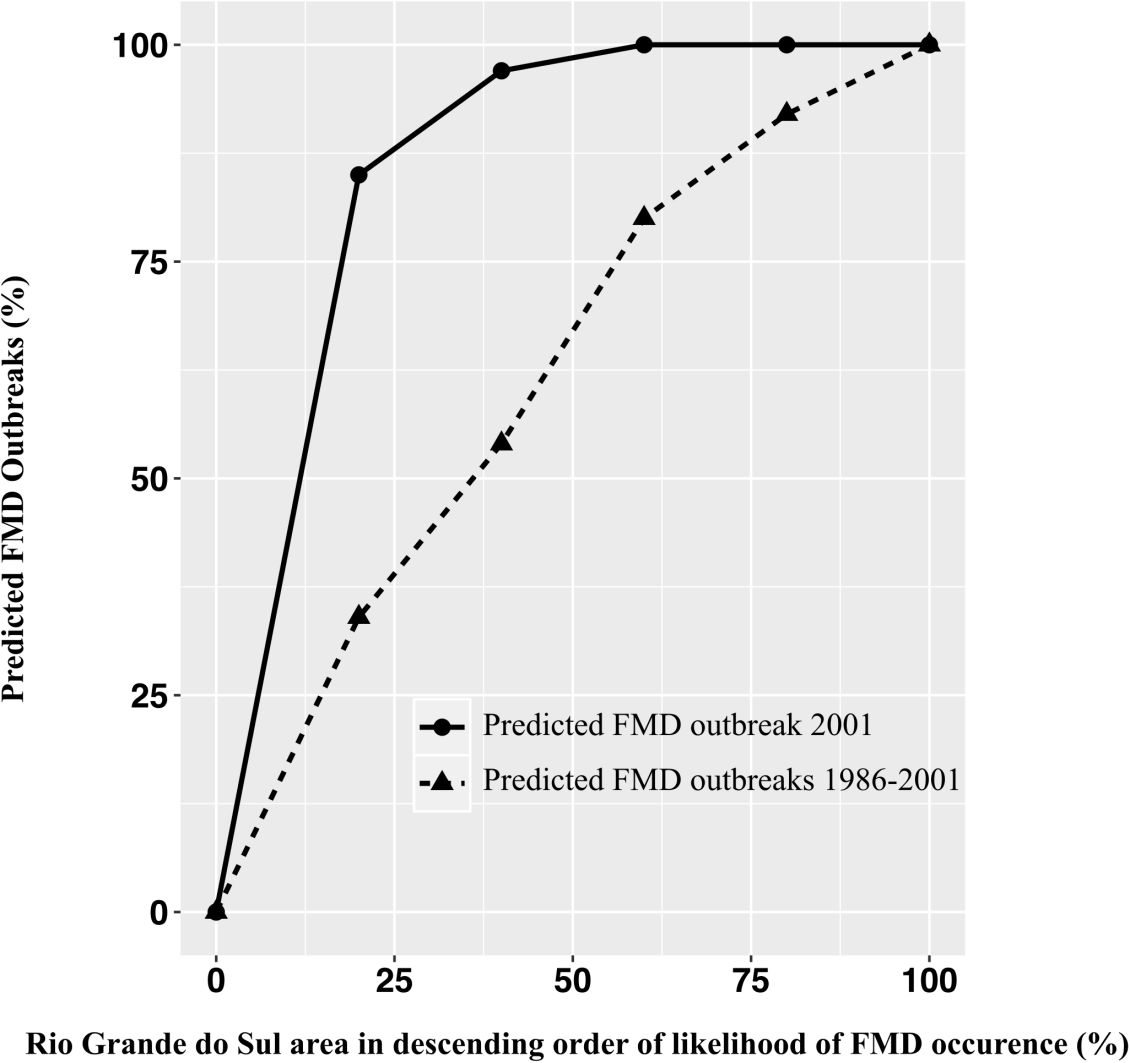
ROC curve for all Rio Grande do Sul FMD outbreaks (1986–2001) and the last outbreak (2001).

From 1986 to 2015, 808 FMD outbreaks were registered in RS. Between 1986 and 1993, 752 FMD outbreaks were registered in all regions of the state, with the highest concentration in the Northwest and Metropolitan mesoregions (Fig 5). Subsequent to this period, RS had two FMD incursions: one in August 2000 with 26 outbreaks and the last in May 2001 with 30 outbreaks (Fig 5). Since 2002, no FMD outbreaks were reported. The AUC value for the historical FMD outbreaks (since 1986) was 0.620 (see S2 File), whereas the AUC for the last FMD outbreak (2001) was 0.864 (see S3 File). The two ROC curves can be visualized in Fig 6.

The map with the identification of FMD high-risk areas in contrast to the 2001 outbreaks of FMD in RS can be observed in Fig 5.

## Discussion

Epidemiological studies can support the OVS on surveillance strategies, decision-making processes and sanitary status improvement [29]. They may also contribute to sounder management of the OVS, allowing safer optimization of resources and increasing public transparency on their activities and responsibilities.

We used the MCDA to create a FMD model that integrates data from diverse sources to identify FMD risk areas in RS. The MCDA has already been employed in other countries [21, 30] and in Brazil [31] to help target animal surveillance and identify risk areas. Our model structure provides a methodology for guiding decision makers throughout the critical process of clarifying evaluation criteria and defining values which are relevant to FMD risk rankings within a defined region. Moreover, it was possible to determine areas where FMD is more likely to be introduced and disseminated to consequently set up or improve target surveillance in these areas to minimize the risk. This type of information is important for the regions that are about to stop the vaccination process, which is the case of the studied region. We found that the likelihood of FMD introduction in RS was strongly associated with proximity to international borders and the density of ruminants such as the Southwest and Southeast mesoregions. Valarcher et al. [15] reviewed all incursions of FMD into Europe between 1985 and 2006 and showed that some FMD outbreaks were associated with close geographical proximity to an international border. Unregulated (illegal or informal) animal movement across international borders is considered a risk associated with FMD introduction and high ruminant density could improve the likely contact with the FMD virus [32]. Other important variables associated with FMD introduction were “density of non-commercial pig farms” and the “count of most intensive surveillance areas”, which were higher in the Northeast and Eastern mesoregions compared with the other mesoregions of the studied area.

Analyzing the likelihood of FMD dissemination in RS, the variables linked to “FMD-susceptible animal movement” had an important participation in the final risk rankings. The majority of annual ruminant movement occurred in the Southwest and Southeast mesoregions, whereas pig movement was concentrated in the Eastern and Northwest mesoregions. In RS the concentration of small farms is greater in the North, which also had the majority of pig and dairy cattle farms, variables associated with FMD dissemination pathways.

The final FMD risk map represented by the likelihood of FMD occurrence showed RS international border areas and the Eastern mesoregion were the areas with the highest FMD areas. Therefore, an OVS plan with FMD target surveillance in these zones would be indicated. The model considered FMD high-risk areas as areas with a likelihood of FMD occurrence within the top 20% among the 1 km x 1 k grids because this cut-off threshold resulted in higher AUC values. However, the best categorization and selection of limits should be carried out by decision makers and the OVS, who may extend or reduce this limit. Given that the improvement of surveillance in large areas needs material and human resources, which are often scarce, the OVS could reallocate these resources from lower FMD risk areas to higher FMD risk areas.

We used the ROC curve as a method to assess the validity of the model by matching data for the FMD high-risk areas identified by the MCDA against FMD outbreaks in RS since 1986. An AUC value of 1 indicates that there is perfect spatial agreement between the class map (FMD high-risk areas) and the historical FMD outbreaks [33]. When analyzing the historical FMD outbreaks since 1986, the model had a moderate performance (0.62). This result could be explained by the fact that one important introduction pathway into RS in the past was associated with laboratory escapes. The metropolitan region used to have five laboratories manipulating the FMD virus for diagnostic and vaccine production, which did not have high biosafety level measures until 1993. Moreover, FMD-infected animals used in laboratories were transported and slaughtered in a neighbouring city approximately 60 km away (J.A. Ravison, personal communication). These procedures without biosafety measures can explain the high concentration of outbreaks in the metropolitan mesoregion, which also led the Brazilian OVS to prohibit the manipulation of the FMD virus in FMD-free zones [20]. Thus, this FMD introduction pathway was evaluated as negligible and not considered in our model. Another factor that might have influenced the moderate performance until 1993 is the fact that FMD outbreak localization was performed using a map grid, which has lower precision compared with the geographic coordinate system adopted from 2000. After 1993, the state did not have FMD outbreaks until 2000. This region was recognized by the OIE as FMD-free with vaccination in May 1998 [3] and subsequently, it had two FMD incursions (Fig 5). When analyzed across the last FMD outbreak in RS, which occurred in 2001, this model was shown to be well adjusted (0.86), and 9 to 10 outbreaks occurred in FMD high-risk areas. This value is similar to other study with animal disease predictive model undertaken in Brazil [34].

The sensitivity analysis was conducted to test the robustness of the results. Changing the weightings of the individual pathways and RF showed that the “proximity to international border” variable affected the mesoregion FMD risk rankings when multiplied by 2. The Northwest mesoregion has the largest international border, whereas the Eastern is localized in the central region (Fig 2) without an international border, and this ranking change was already expected.

This study also evaluated the FMD surveillance performance applied in RS related to the FMD high-risk areas. Fortunately, in the Southwest and Southeast regions, which were the top in terms of the FMD risk rankings, the FMD surveillance performance was the best compared with the other regions (Table 5). However, the Eastern and Northwest regions, which also had FMD high-risk areas, need to improve their FMD SP. Animal surveillance has been associated with stakeholders and their participation in the surveillance process is very important [35]. The analysis of FMD surveillance performance variables showed that the “presence of OVS” together with the “number of FMD-susceptible animal disease investigations reports” were the most relevant variables considered by the FMD experts. Although important communication tools such as the Internet and mobile phones are currently available [36], the physical presence and proximity of a veterinary government office are important to farmers, especially for disease notification by small farmers. In RS, personal notifications are the main form of contact between farmers and OVS [37]. The number of animal disease investigations is associated directly with the reports made by the farmers [10]. Regions which have more FMD-susceptible animal disease investigation reports could be associated with greater community participation in animal health issues; therefore, they can be quickly informed of a possible FMD introduction. Other investigations (the number of non-FMD-susceptible animals), despite not being associated with FMD disease, can indirectly measure community participation in aspects linked to animal health. It is valuable to highlight that the SP_index_ has a limitation in this study. The “private veterinarian density” variable was used; however, this data source does not have accurate information since a professional veterinary can work in all state areas. Furthermore, we did not have information about the nature of field work carried out by veterinarians. In order to calculate the SP_index_, the number of private veterinarians registered in each city was used, without knowing the proportion of those that may work in areas not related to the livestock sector.

For future analyses, we recommend this model to be frequently updated because all data are available at the RS OVS, as well as technological tools can be used to improve the systematization of this multi-criteria model. Additionally, georeferenced data of RS farms could be adopted, when available, to replace the variables used as polygons (only information of city), which would make the model more accurate. Environment variables (e.g., forest areas, hills, valleys) could be considered natural barriers to FMD introduction and dissemination and they can be contemplated to improve the precision of the model.

Furthermore, temporal components could be useful if combined with spatial risk model, so as to improve the results. It is possible that there are times of the year (e.g. the coolest months) when FMD virus is more likely to survive and spread. Moreover, in the present study, only the “presence of OVS” was examined, but road layers can be added to the model to measure the farm-office distance to improve the accuracy of the model. It is important that future studies complement the SP_index_ by improving type of work carried out by private veterinarians and adding new variables and inserting other diseases. Then, a single index could be created, as realized by East et al. [10], which would help the RS OVS to improve the animal surveillance performance.

Concerning data analysis, whenever the data is not available, dynamical models are useful in the analysis of infectious disease, such as deterministic models [38–42]. These models can aid identifying risk areas based on the dynamic of occurrence of diseases in a given area according with some parameters that can vary depending on its geolocation.

Our study utilized a robust South American expert panel to define the weighing of the pathways and associated risk factors that were essential to the final model. This process had 13 participants from five South American nationalities with great knowledge about FMD disease in this region. We had an excellent response rate of 52%, which helped to determine the weight of each pathway and the risk factors used in the model. Garabed et al. [43] used a similar eliciting process and obtained a response rate of 16%. The AHP have been used in other studies [44] and it was the key to success in the eliciting process because it quickly showed the consistency of the responses. Conducting the eliciting process by email had some advantages when compared with group meetings because it prevented any interaction or exchange of information between the participants. On the other hand, the Delphi approach did not produce good results. Cox et al. [25] utilized a Delphi-like approach and obtained only 4 experts (out of 64) that altered their responses, which is a rate similar to the one found in this study. Contact via email and the repetitive and time-consuming nature of the procedure may explain the lack of response [25].

South American areas have similar animal production characteristics and this model, with some adjustments, could be used in other areas of the continent. The main objective of the MCDA in supporting spatial decision making is to help the decision participants to develop a constructive and creative approach to the problem at hand, rather than to support them in identifying the ‘best’ solution [45]. Likewise, in several states in Brazil, the main actors involved with livestock activities (stakeholder farmers, government, and the cattle, sheep, and swine industries) have discussed the possibility of a forthcoming stoppage of vaccination. In areas without FMD vaccination, the risk of FMD establishment and spread is higher than in zones with FMD vaccination. Brazilian states classified as FMD-free areas with vaccination could apply this model to evaluate and, if necessary, improve surveillance actions in FMD high-risk areas to change to FMD-free areas without vaccination with higher security. Therefore, surveillance measures might be taken by the OVS to minimize those risks in the FMD high-risk areas.

## Supporting information

S1 File Table A and scenario tree. (DOCX)

S2 File Data of the ROC curve for the historical FMD outbreaks. (TXT)

S3 File Data of the ROC curve for the last FMD outbreak. (TXT)
