# Supplementary material for: Identification of foot and mouth disease risk areas using a multi-criteria analysis approach
Source: PLoS One. 2017 May 26;12(5):e0178464. doi: 10.1371/journal.pone.0178464 (PMC5446179; doi:10.1371/journal.pone.0178464)
Supplement: S1 File — (DOCX) [file pone.0178464.s001.docx]

**S1 - Supporting Information**

### Data Layers

**Table A** – The sources of information for all variables used to prepare raster map layers are shown in Table A.

### Scenario Tree

**Scenario tree** showing FMD introduction and exposure pathways in Rio Grande do Sul, Brazil

**Table A** – Variables used in the model to prepare raster map layers.

| **Variable** | **Weight*** | **Description** | **Source** |
| --- | --- | --- | --- |
| International border proximity | 16.00 | Raster created by Idrisi for international border (Argentina and Uruguay). Distance ranging from 0 to 50 km from international borders. |  |
| Ruminant density | 12.19 | Density of ruminant in county (number of ruminants/km^2^) | SAN, 2013 |
| Non-commercial pig farm density | 10.59 | Density of farms with pigs for non-commercial usage in county (number of non-commercial pig farms/km^2^) | SAN, 2013 |
| Most intensive surveillance area count | 9.00 | Numbers of land reform farms, “quilombola” communities, indigenous areas, sanitary landfills and dumping grounds in county | SEAPI, unpublished results |
| Pig movement for all purposes, except slaughter | 6.85 | Number of load of pigs transported between counties annually for all purposes except slaughter (number of load of pigs/year) | SAN, 2013 |
| Ruminant movement for all purposes except animal fair and slaughter | 6.54 | Number of load of ruminants transported between counties annually for all purposes except animal fair and/or slaughter (number of load of ruminants/year) | SAN, 2013 |
| Environmental suitability for FMD virus | 5.76 | Annual count relative to monthly average humidity of greater than 60% | CPTEC, 2014 |
| Bovine and buffalo count | 5.71 | Domestic cattle and buffalo number in county (number of animals) | SAN, 2013 |
| Ruminant movement to animal fairs | 5.67 | Number of load of ruminants transported annually to markets and shows (number of load of ruminants/year) | SAN, 2013 |
| FMD-susceptible animal farm density | 4.05 | Density of farms with at least one susceptible animal in (each) county (number of farms/km^2^) | SAN, 2013 |
| Pig farm density | 3.63 | Density of pig farms in county (number of pig farms/km^2^) | SAN, 2013 |
| FMD-susceptible animal movement to slaughterhouse | 3.44 | Number of load of FMD-susceptible animal transported annually to slaughterhouses in county (number of FMD-susceptible animals movement /year) | SAN, 2013 |
| International port and airport presence | 2.84 | Counties with an international airport and port with high movement of products and people were assigned the maximum value (1). Scores were adjusted according to this base for all other counties. | INFRAERO, 2013; SPH, 2013 |
| Dairy farm, reproduction cattle farm and commercial pig farm density | 2.74 | Density of dairy farms, reproduction cattle farms and commercial pig farms in county (number of farms/km^2^) | SAN, 2013 |
| Presence of international farm fair and waiting place for cattle export | 2.01 | Counties that have an international farm fair and waiting place for cattle export with annual high animal movement were assigned the maximum value (1), and scores were adjusted according to this base for all other counties. | SEAPI unpublished results |
| Ruminant farm density | 1.53 | Density of ruminant farms in county (number of ruminant farms/km^2^) | SAN, 2013 |
| Wild boar area proximity | 0.74 | Buffer to wild boar area. Distance ranging from 0 to 60 km (wild boar range of movement). | Medeiros, 2016 |
| Commercial pig farm density | 0.74 | Commercial pig farm density in county (number of commercial pig farms/km^2^) | SAN, 2013 |
| Private veterinarian density | - | Private veterinarian density in county (number of private veterinarian offices/km^2^) | CMRV/RS, Unpublished results |
| FMD-susceptible animal disease investigation reports | - | Annual median number of FMD-susceptible animal disease investigations in county in 2012, 2013 and 2014 divided by the total number of susceptible animal farms in county (FMD-susceptible animal disease investigation median/total susceptible farms) | SEAPI, 2013 |
| Non-FMD-susceptible animal disease investigation reports | - | Annual median number of non-FMD-susceptible animal disease investigations in county in 2012, 2013 and 2014 divided by the total number of farms in county (non-FMD-susceptible animal disease investigation median/total farms) | SEAPI, 2013 |
| Veterinary office presence | - | Counties with government veterinary office presence were assigned a value of 1; those with only a government veterinary community (inside other governmental institutions) were assigned a value of 0.5; and those without a government veterinary office or room were assigned a value of 0. | SAN, 2013 |

* Variable participation percentage in the likelihood of FMD occurrence

**Variable types**: The variables “international border proximity” and “wild boar area proximity” were developed from points to the raster layer. All other variables were developed from data from counties (polygon) to the raster layer.

#

# References

CPTEC, 2014: Centro de Previsão de Tempo e Estudos Climáticos. Available at: <http://bancodedados.cptec.inpe.br/> (Accessed 8 April 2015).

INFRAERO, 2013: Empresa Brasileira de Infraestrutura Aeroportuária. Available at: <http://www.infraero.gov.br/portal/index.php/us.html> (Accessed 11 June 2014).

Medeiros, A.A.R. 2016. Avaliação dos fatores associados ao contato entre javalis asselvajados e suínos de subsistência no Rio Grande do Sul. MSc thesis, UFRGS, Porto Alegre, Brazil.

SAN, 2013: Animal data analysis systems of the Rio Grande do Sul Official Veterinary Service. Only access to OVS members and Rio Grande do Sul farmers. Available at: <http://www.dda.agricultura.rs.gov.br/conteudo/6433/?SDA_-_Sistemas_de_Defesa_Agropecuária>(Accessed 3 – 7 March 2014).

SEAPI, 2013: Rio Grande do Sul Official Veterinary Service. Available at: [http://www.dda.agricultura.rs.gov.br/lista/977/Se%C3%A7%C3%A3o_de_Epidemiologia_e_Estat%C3%Adstica](http://www.dda.agricultura.rs.gov.br/lista/977/Se%C3%A7%C3%A3o_de_Epidemiologia_e_Estat%C3%ADstica) (Accessed 10 March 2014).

SPH, 2013: Superintendencia de Portos e Hidrovias. Available at: <http://www.sph.rs.gov.br/sph_2006/content/localizacao/localizacao.php> (Accessed 11 June 2014).

**Scenario tree –** FMD introduction and exposure pathways in Rio Grande do Sul, Brazil

**Definitions and Labels**

**Definition:**

1. Introduction module:
2. Introduction – entry of the FMD virus in Rio Grande do Sul.
3. Exposure – infection of the first FMD-susceptible animal (quantity of virus that caused first animal infection)
4. Dissemination module:
5. Establishment – infection of the first group (herd) of FMD-susceptible animals by windborne spread, direct contact or fomites/products/vehicles
6. Spread – infection of the second and subsequent groups of FMD-susceptible animals by windborne spread, direct contact or fomites/products/vehicles

**Labels:**

| Decision node |
| --- |
|  |
| No FMD Outbreak |
| FMD Outbreak |
| ------- FMD Negligible likely pathway |
|  |
| _____ **FMD Non-Negligible likely pathway** |

**Scenario tree –** FMD introduction and exposure pathways in Rio Grande do Sul, Brazil (1/6)

**Primary introduction – Pathways**

1. **Fomites/Bioterrorism**

--------------------------------- (2) FMD vaccine

Foot and Mouth Virus (3) **Animal products**

--------------------------------- (4) Laboratory (FMD virus escape)

(5) **Live FMD-susceptible domestic animals**

**Scenario tree –** FMD introduction and exposure pathways in Rio Grande do Sul, Brazil (2/6)

**Pathway 1** – Introduction FMD virus at fomites/bioterrorism

FMD introduction and exposure pathway in Rio Grande do Sul, Brazil

**Exposure to FMD-**

**susceptible animals** **FMD**

**Outbreak**

**Not detected by**

**FMD surveillance** **Contact with** No exposure to FMD-

**FMD-susceptible animals** susceptible animals No FMD

No contact with Outbreak

FMD-susceptible animals No FMD

Introduction FMD virus at Outbreak

fomites/people/vehicles **Bioterrorism**
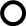
 Detected by FMD surveillance No FMD

(voluntary) Outbreak

Quarantine and biosecurity No FMD

measures to detect and eliminate FMD virus Outbreak

**Fomites**  **FMD virus remains viable after** **Exposure to FMD-** **FMD**

(Veterinarians, farmers, animal food,  **biosecurity and quarantine measures** **susceptible animals** **Outbreak**

equipment, vehicles, etc.)

No exposure to FMD-


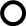
 **Contact with** susceptible animals No FMD

**FMD-susceptible animals** Outbreak

No contact with No FMD

FMD-susceptible animals Outbreak

**Scenario tree -** FMD introduction and exposure pathways in Rio Grande do Sul, Brazil (3/6)

**Pathway 2 –** FMD virus-contaminated vaccine

FMD introduction and exposure pathway in Rio Grande do Sul, Brazil

-------- Exposure to FMD- -------- FMD

susceptible animals Outbreak

No exposure to FMD- No FMD

Inefficiently inactivated -----
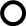
 ------------------ Contact with ------- susceptible animals Outbreak

FMD vaccine FMD-susceptible animals

No contact with No FMD

FMD-susceptible animals Outbreak

FMD vaccine **------------** Free zone with FMD -------------
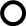


vaccination maintenance

Inactivated FMD vaccine No FMD

Outbreak

FMD free without vaccination No FMD

Outbreak

**Scenario tree -** FMD introduction and exposure pathways in Rio Grande do Sul, Brazil (4/6)

**Pathway 3 –** FMD virus-contaminated animal products

FMD introduction and exposure pathway in Rio Grande do Sul, Brazil

Exposure to FMD-

susceptible animals -------------------------- **FMD**

**Outbreak**

FMD virus remains viable after

biosecurity and quarantine --------- ---------------- Contact with ------ No exposure to FMD- No FMD

measures FMD-susceptible animals susceptible animals Outbreak

No contact with No FMD

FMD-susceptible animals Outbreak

No FMD

Quarantine and biosecurity Outbreak

---------Legal ---------------
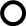
 measures detect and eliminate FMD virus

importation

FMD virus-contaminated
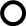
 No FMD

animal products No contact with Outbreak

FMD-susceptible animals

**Illegal** No exposure to FMD- No FMD

**importation**
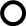
 **Contact with FMD-**
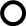
 susceptible animals Outbreak

**susceptible animals**

**Exposure to FMD-** **FMD**

**susceptible animals** **Outbreak**

**Scenario tree -** FMD Introduction and Exposure pathways in Rio Grande do Sul, Brazil (5/6)

**Pathways 4 –** Escape of FMD virus from laboratory

FMD introduction and exposure pathway in Rio Grande do Sul, Brazil

There are no laboratories with No FMD

FMD virus manipulation Outbreak

Animal laboratory
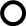
 ------------- There are laboratories with

FMD virus manipulation

(FMD diagnostic or

FMD vaccine production)

Laboratories have appropriate No FMD


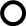
 biosecurity level Outbreak

Exposure to FMD- FMD

susceptible animals -------------------- outbreak

Lab biosecurity failure --------- ---------------- Contact with ----- No exposure to FMD- No FMD

FMD virus escape FMD-susceptible animals susceptible animals Outbreak

No contact with No FMD

FMD-susceptible animals Outbreak

No FMD

**Scenario tree -** FMD introduction and exposure pathways in Rio Grande do Sul, Brazil (6/6)

**Pathway 5 –** Contamination of live animals by FMD virus

FMD introduction and exposure pathway in Rio Grande do Sul, Brazil

No FMD

No contact with Outbreak

FMD-susceptible animals

Free-living No exposure to FMD- No FMD

wild animals
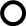
 **Contact with FMD-**
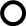
 susceptible animals Outbreak

**susceptible animals**

**Exposure to FMD-** **FMD**

**susceptible animals** **Outbreak**

---------------------------- FMD-susceptible **Illegal animal** Quarantine or clinic and laboratory No FMD

Wild animals **movement** tests detect and eliminate FMD virus Outbreak

aptivity of Legal animal

**FMD virus-contaminated** wild animals
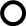
 ---------- movement

**live animals**

**FMD-susceptible**  ------- Exposure to FMD--------- **FMD**

**domestic animals** Quarantine or clinic and laboratory susceptible animals **Outbreak** tests do not detect or eliminate FMD virus

------------- Contact with ------- No exposure to FMD- No FMD

FMD-susceptible animals susceptible animals Outbreak

No contact with

FMD-susceptible animals No FMD

Outbreak

----------------------------Other animals

(FMD virus carriers)
